# Supplementary material for: Surveillance-embedded genomic outbreak resolution of methicillin-susceptible Staphylococcus aureus in a neonatal intensive care unit
Source: Sci Rep. 2020 Feb 14;10:2619. doi: 10.1038/s41598-020-59015-1 (PMC7021795; doi:10.1038/s41598-020-59015-1)
Supplement: Supplementary file 1 — Supplementary Material. [file 41598_2020_59015_MOESM1_ESM.pdf]

Surveillance-embedded genomic outbreak resolution of methicillin-susceptible *Staphylococcus aureus* in a neonatal intensive care unit

AJH Cremers[1‡*], JPM Coolen[1‡], CP Bleeker-Rovers[2], ADJ van der Geest-Blankert[3], D Haverkate[1], H Hendriks[4], SSV Henriet[5], MA Huynen[6], E Kolwijck[1], D Liem[4], WJG Melchers[1], JW Rossen[7], J Zoll[1], A van Heijst[4], J Hopman[1#], HFL Wertheim[1#]

[1]Department of Medical Microbiology, Radboudumc center for infectious diseases, Nijmegen, the Netherlands

[2]Department of Internal Medicine, Radboudumc center for infectious diseases, Nijmegen, the Netherlands

[3]Occupational Health & Safety and Environmental Service, Radboudumc, Nijmegen, the Netherlands

[4]Department of Neonatology, Radboudumc, Nijmegen, the Netherlands

[5]Department of Pediatrics, Radboudumc Amalia Children's Hospital, Nijmegen, the Netherlands

[6]Centre for Molecular and Biomolecular Informatics, Radboud Institute for Molecular Life Sciences, Radboudumc, Nijmegen, the Netherlands

[7]Department of Medical Microbiology and Infection Prevention, University of Groningen, University Medical Center Groningen, 9713 GZ Groningen, the Netherlands.

[‡] AC and JC contributed equally to this manuscript.

[#] JH and HW contributed equally to this manuscript.

* Corresponding author: AJH Cremers; email: amelieke.cremers@radboudumc.nl; tel: +31-24-3619041.

Keywords: MSSA, outbreak, genomics, NICU.

Supplementary Material

Supplementary Methods

Supplementary Methods 1. Definition of MSSA infection.

In case a neonate was suspected for a respiratory infection, a standard work-up including blood cultures was performed. Respiratory specimens were not collected for surveillance purposes, yet only when a respiratory infection was suspected. If a respiratory specimen was the most invasive material in which *S. aureus* had been identified, we retrospectively stratified the likelihood of a true *S. aureus* infection to be unlikely, possible, or probable.

S. aureus infection was denominated unlikely if a respiratory *S. aureus* infection was not adequately covered by a subsequently administered antibiotic treatment regimen, if a more evident infectious cause was identified, if clinical signs at specimen collection were deemed unrelated to infection based upon the subsequent clinical course, or if the culture result was explicitly considered as not related to infection by the treating physician. Unlikely cases were removed from further analyses of *S. aureus* infections. Among the remaining cases with positive respiratory specimens *S. aureus* infection was denominated probable if the neonate was in respiratory distress, and had a blood CRP-level >20mg/L or a lung infiltrate on chest X-ray. The remaining cases were classified as possible *S. aureus* infection.

Supplementary Methods 2. WGS methods.

DNA isolation and library preparation

Selected MSSA isolates were cultured overnight at 36°C on BD trypticase soy agar II with 5% sheep blood. Bacterial DNA was extracted by a CTAB-based method. Colonies were resuspended in 400 µL TE buffer (10 mM Tris pH8.0, 1 mM EDTA) and 50 µL of 10 mg/mL lysozyme was added. Samples were incubated for 60 min at 37°C, whereafter 75 µL of 0.7 mg/mL proteinase K in 10% SDS was added followed by an incubation for 10 min at 65°C. The samples were mixed with 100 µL of 5M NaCl and 100 µL CTAB/NaCl solution (1% N-cetyl-N,N,N,-trimethyl ammonium bromide in 0.7 M NaCl) and incubated for 10 min at 65°C. DNA was further isolated using chloroform/isoamylalcohol extraction. DNA was precipitated from the aqueous phase by adding an equal amount of 2-propanol and subsequent incubation for 20 min at -20°C. Samples were centrifuged for 10 min at 11.000*g*. DNA pellets were washed with 1 mL cold 75% ethanol and centrifuged for 5 min at 11.000*g*. DNA pellets were air-dried for 15 min at room temperature and dissolved in 100 µL TE buffer. DNA samples were quantified using the QuantiFluor dsDNA system (Promega, Madison, WI, USA). A fragmented genomic DNA library was prepared using a NexteraXT DNA sample preparation kit (Illumina, San Diego, CA, USA). Subsequent sequencing was conducted in a paired-end 2 x 150bp mode using an Illumina NextSeq500 sequencer (Illumina, San Diego, CA, USA).

Read trimming and assembly

Sequence reads containing Nextera XT adapters and low quality regions were detected and removed by Trim_galore (version 0.4.1) (1), using *"--length 150 --stringency 12"* settings. Reads with tailing artefacts (stretches of minimally 6 A's or 6 G's introduced by sequencing chemistry) were removed using a custom python script. Reads with lengths of either 150 or 151 were used for de novo assembly. Prior to assembly the read coverage was estimated by dividing the number of nucleotides on the filtered reads by the length of *Staphylococcus aureus* strain RIVM1295 (RefSeq:NZ_CP013616.1). Reads were randomly subsampled to 120x coverage if coverage exceeded. To obtain high quality assemblies, isolates with an average coverage < 20x were discarded from further analyses. Reads were assembled into contigs with SPAdes 3.10.1 (2) at default settings and using k-mer sizes 21, 41, 61, 81 and 101. Contigs were annotated using PROKKA (3). Read depth at each position was determined by mapping reads to the contigs using Bowtie2 (version 2.2.9) (4) *"--no-unal –no-discordant"*, followed by Samtools (version 1.3.1) (5) depth estimation. The Percentage of reads mapped to the contigs is used as measure of

assembly quality. Prior to outbreak analyses contigs with a mean coverage depth < 20x and/or contigs with length < 500 bp were discarded.

SNP handling

Single nucleotide polymorphisms (SNPs) were determined by k-mer extraction from qualified contigs, using kSNP (version 3.021) (6). The optimal k-mer size for *S. aureus* of 19bp was determined using Kchooser (6). High quality SNPs (hqSNPs) were obtained by mapping the k-mers to the contigs using Bowtie2 (4) "--all". k-mers that map perfectly multiple times and/or not map perfectly were excluded for further analyses. A custom python code derived each k-mer location from the bam files and determines the exact SNP position on the contigs. Prokka annotation file in gff format is parsed to match the SNP location, adds annotation to be able to determine if SNP location is on coding or non-coding region of the genome. Previously determined read depth is used to filter SNPs with ≥ 20x read depth, resulting in hqSNPs. Core hqSNPs were defined as hqSNP positions located at genomic regions that were present in all strains selected for a particular analysis. Phylogeny by a maximum likelihood tree was inferred from all concatenated core hqSNPs per strain. The model was determined by using jModelTest 2.1.10 (7) resulting by applying PhylML (8) with custom best model "-d nt -n 1 -b 0 -m 012314 -f m -c 1 --no_memory_check -o tlr -s BEST". Visualization of the phylogenetic tree was done using iTOL v4.1.1 (9). Kruskal's Minimum Spanning Trees (MST) (10) were calculated from pairwise core hqSNP differences between all strains, using networkx 1.11 in Python 2.7.5, and visualized using Cytoscape (3.5.1) (11).

Supplementary Tables

Supplementary Table 1. Monthly results throat swab surveillance.

Year	Month	Number collected	Number positive	Percentage positive
**2014**	**Jan**	61	9	15
	**Feb**	44	6	14
	**Mar**	54	9	17
	**Apr**	49	15	31
	**May**	47	19	40
	**Jun**	54	11	20
	**Jul**	39	3	8
	**Aug**	40	7	18
	**Sep**	59	14	24
	**Oct**	45	5	11
	**Nov**	48	9	19
	**Dec**	58	17	29
**2015**	**Jan**	39	3	8
	**Feb**	43	1	2
	**Mar**	53	4	8
	**Apr**	37	5	14
	**May**	47	5	11
	**Jun**	51	11	22
	**Jul**	52	5	10
	**Aug**	57	17	30
	**Sep**	46	19	41
	**Oct**	40	9	23
	**Nov**	45	7	16
	**Dec**	46	4	9

<u>Supplementary Table 2.</u> Neonatal MSSA infections and carriage.

	**INFECTED**			**CARRIERS**		
	**All (n=40)**	**2014 (n=27)**	**2015 (n=13)**	**All (n=100)**	**2014 (n=55)**	**2015 (n=45)**
**Age (days)**	18 (9-28)	18 (9-23)	21 (11-33)	4 (1-5)	4 (1-5)	4 (1-5)
**Healthcare associated**[a]	93% (37/40)	93% (25/27)	92% (12/13)			
**Carrier (n/n tested)**	70% (28/40)	78% (21/27)	54% (7/13)	20% (100/510)	22% (55/256)	18% (45/254)
**Times screened for carriage (including throat and anal swabs)**	11 (6-13)	10 (6-12)	12 (6-14)	1 (1-3)	1 (1-3)	1 (1-3)
**Sterile site**	17	12	5			
HA-BSI	*12*	*9*	*3*			
CA-BSI	*3*	*2*	*1*			
Cerebrospinal fluid	*1*	*1*	*0*			
Pleural fluid	*1*	*0*	*1*			
**Non sterile site**	23	15	8			
Respiratory specimen	*13*	*9*	*4*			
Eye	*5*	*3*	*2*			
Wound	*2*	*1*	*1*			
Navel	*1*	*1*	*0*			
Skin	*2*	*1*	*1*			
**Spa clustering (n cases/n individual spa types)**	2.1 (32/15)	2.1 (21/10)	1.6 (11/7)	2.5 (80/32)	2.2 (39/18)	1.9 (41/22)
**Outbreak-MLVA associated**	40% (14/35)	42% (10/24)	36% (4/11)	63% (20/32)	55% (12/22)	80% (8/10)
MT0007	*4*	*3*	*1*	*5*	*1*	*4*
MT0388	*3*	*3*		*7*	*7*	
MT0005	*2*	*2*				
MT1905	*2*	*2*		*4*	*4*	
MT0368	*3*		*3*	*4*		*4*

[a]Onset after 48 h of hospitalization or after 48 h postnatally in neonates not discharged from hospital.

Abbreviations: HA-BSI: Healthcare associated blood stream infection; CA-BSI: Community acquired blood stream infection; *Spa*: Staphylococcal protein A; MLVA: Multi locus variant analysis; MT: MLVA type.

Supplementary Table 3. hqSNP locations between isolates RUMC_0017 and RUMC_0071.

**Contig ID**[a]	**SNP location**	**Gene**[b]	**Product**[b]
contig_1_281813_52.1041			
	19437	rplI	50S ribosomal protein L9
	143138	bceB_3	Bacitracin export permease protein BceB
	151210	Intergenic region	Intergenic region
	187821	yciC_2	Putative metal chaperone YciC
	267482	zntR	HTH-type transcriptional regulator ZntR
contig_2_205801_37.4917			
	106370	ureB	Urease subunit beta
	127687	NA	Putative formate dehydrogenase
	146426	yghA	putative oxidoreductase YghA
contig_3_191392_27.4066			
	161082	pepT_2	Peptidase T
contig_4_187726_53.7231			
	31825	aldA	Putative aldehyde dehydrogenase AldA
	78744	NA	hypothetical protein
	83823	Intergenic region[c]	Intergenic region[c]
	123321	ptsG_2	PTS system glucose-specific EIICBA component
contig_5_164670_42.7028			
	64051	Intergenic region[c]	Intergenic region[c]
	83142	gltB_1	Glutamate synthase [NADPH] large chain
	88439	pbuE	Purine efflux pump PbuE
contig_6_157929_28.8239			
	41880	pnp	Polyribonucleotide nucleotidyltransferase
	124088	carB	Carbamoyl-phosphate synthase large chain
	136277	ileS	Isoleucine--tRNA ligase
contig_8_116174_34.7802			
	12685	pgk	Phosphoglycerate kinase
	60881	nrdF	Ribonucleoside-diphosphate reductase subunit beta
contig_9_115158_29.0769			
	27916	atl_2	Bifunctional autolysin
	34247	sspA_1	Glutamyl endopeptidase
	55094	yfkN_2	Trifunctional nucleotide phosphoesterase protein YfkN
	113184	yitU_2	5-amino-6-(5-phospho-D-ribitylamino)uracil phosphatase YitU
contig_13_81994_34.3325			
	36150	NA	hypothetical protein
contig_16_68550_30.8619			
	34455	dinB	DNA polymerase IV
	44517	vraS	Sensor protein VraS
contig_18_62260_50.3488			
	15161	btrK	L-glutamyl-[BtrI acyl-carrier protein] decarboxylase
contig_19_48730_42.3991			
	28563	NA	hypothetical protein
	32206	NA	Glycyl-glycine endopeptidase ALE-1
contig_20_41580_60.8721			
	22151	metQ_2	putative D-methionine-binding lipoprotein MetQ
contig_21_40230_32.6075			
	6599	Intergenic region[c]	Intergenic region[c]
	29657	fumC	Fumarate hydratase class II
contig_22_39715_38.5618			
	17835	nrgA	Ammonium transporter NrgA
contig_23_39056_48.9101			
	16031	glmU	Bifunctional protein GlmU
contig_24_36307_42.5898			
	21503	ylmA	putative ABC transporter ATP-binding protein YlmA
	30152	gmuF	putative mannose-6-phosphate isomerase GmuF

contig_25_35998_38.4754			
	10089	thrB_2	Homoserine kinase
	24548	yhfT	putative acyl--CoA ligase YhfT
	34373	gtf1_2	Glycosyltransferase Gtf1

[a]Contig ID as generated by SPAdes.
[b]Annotation as determined by PROKKA.
[c]SNP located on region with no gene as predicted by PROKKA.

Supplementary Figures

Supplementary Figure 1. Flow chart screening HCWs.

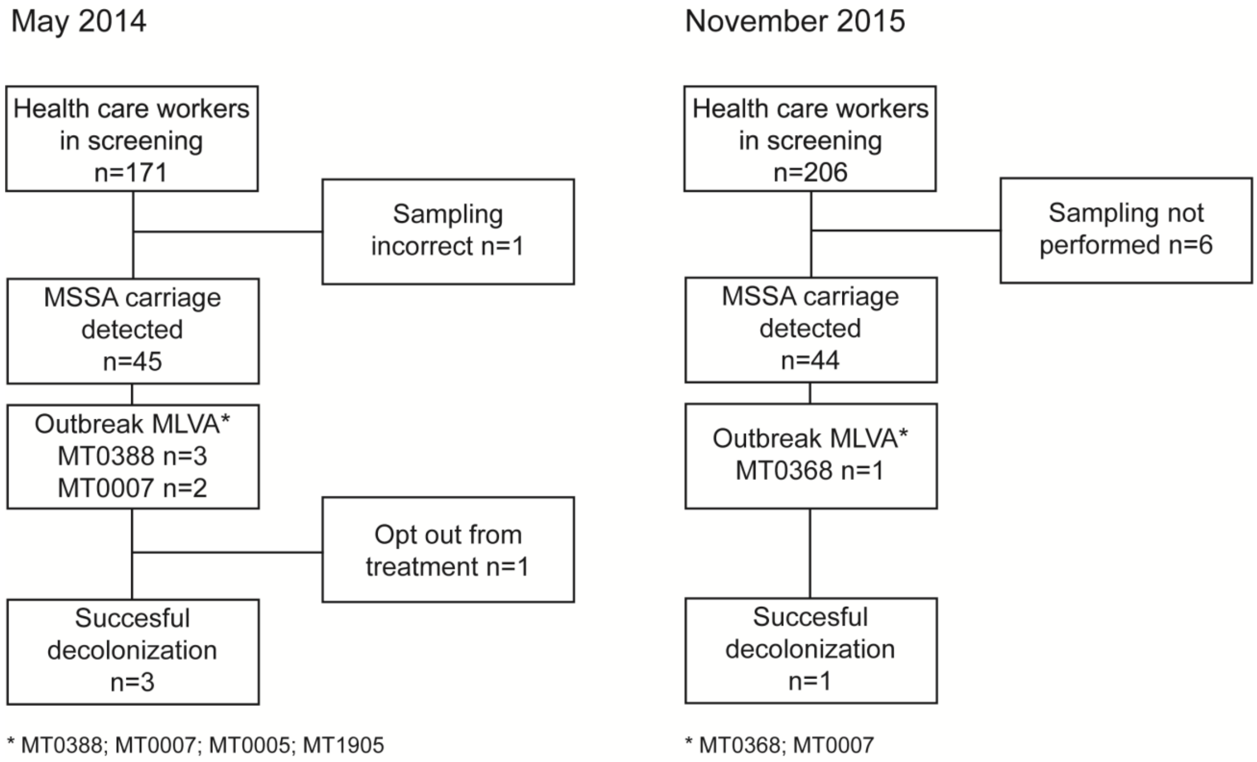

Supplementary Figure 2. Diversity in percentages of pairwise core hqSNPs between members of particular *spa* types.

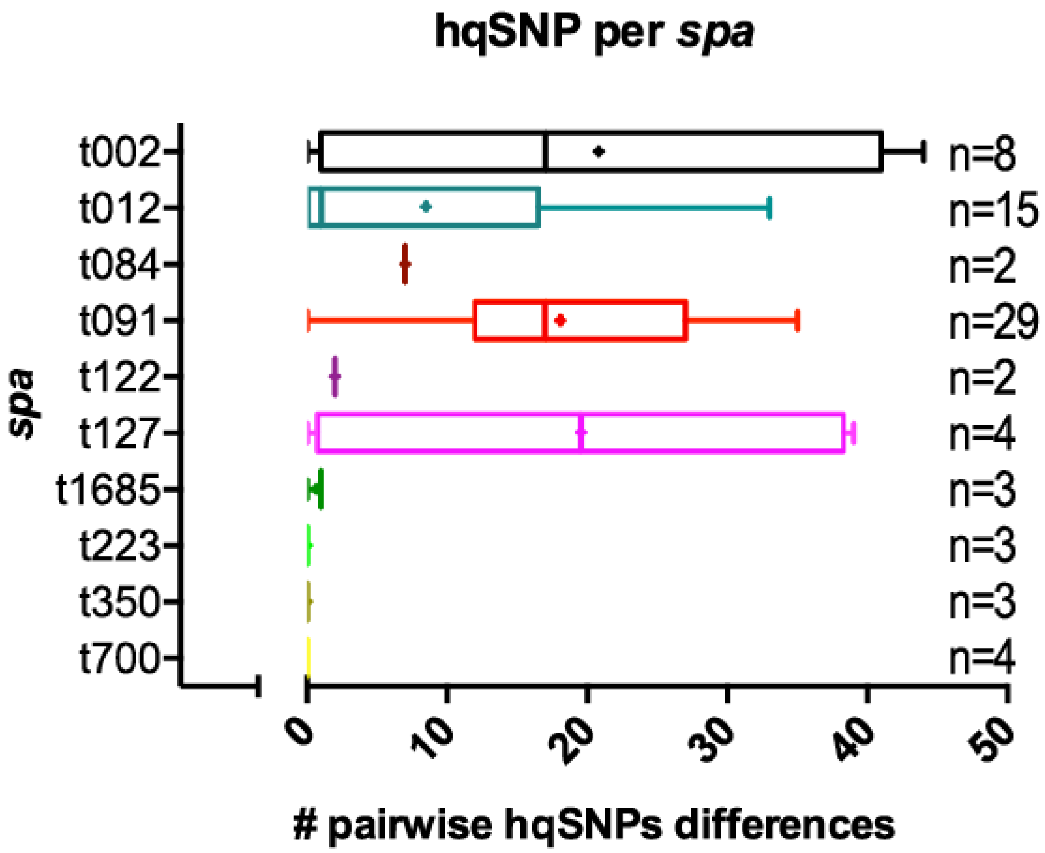

References

1. Martin M. CUTADAPT removes adapter sequences from high-throughput sequencing reads2011.
2. Bankevich A, Nurk S, Antipov D, Gurevich AA, Dvorkin M, Kulikov AS, et al. SPAdes: a new genome assembly algorithm and its applications to single-cell sequencing. J Comput Biol. 2012;19(5):455-77.
3. Seemann T. Prokka: rapid prokaryotic genome annotation. Bioinformatics. 2014;30(14):2068-9.
4. Langmead B, Salzberg SL. Fast gapped-read alignment with Bowtie 2. Nat Methods. 2012;9(4):357-9.
5. Li H, Handsaker B, Wysoker A, Fennell T, Ruan J, Homer N, et al. The Sequence Alignment/Map format and SAMtools. Bioinformatics. 2009;25(16):2078-9.
6. Gardner SN, Slezak T, Hall BG. kSNP3.0: SNP detection and phylogenetic analysis of genomes without genome alignment or reference genome. Bioinformatics. 2015;31(17):2877-8.
7. Darriba D, Taboada GL, Doallo R, Posada D. jModelTest 2: more models, new heuristics and parallel computing. Nat Methods. 2012;9(8):772.
8. Guindon S, Gascuel O. A simple, fast, and accurate algorithm to estimate large phylogenies by maximum likelihood. Syst Biol. 2003;52(5):696-704.
9. Letunic I, Bork P. Interactive tree of life (iTOL) v3: an online tool for the display and annotation of phylogenetic and other trees. Nucleic Acids Res. 2016;44(W1):W242-5.
10. Kruskal JB. On the Shortest Spanning Subtree of a Graph and the Traveling Salesman Problem. Proceedings of the American Mathematical Society. 1956;7(1):48-50.
11. Shannon P, Markiel A, Ozier O, Baliga NS, Wang JT, Ramage D, et al. Cytoscape: a software environment for integrated models of biomolecular interaction networks. Genome Res. 2003;13(11):2498-504.
